# Retraction Note to: Fungal sensing skin

**DOI:** 10.1186/s40694-021-00114-7

**Published:** 2021-06-02

**Authors:** Andrew Adamatzky, Antoni Gandia, Alessandro Chiolerio

**Affiliations:** 1 Unconventional Computing Laboratory, UWE, Bristol, UK; 2 Mogu S.r.l, Inarzo, Italy; 3 Center for Sustainable Future Technologies, Istituto Italiano di Tecnologia, Torino, Italy

## Retraction Note to: Fungal Biol Biotechnol (2021) 8:3 https://doi.org/10.1186/s40694-021-00110-x

The Publisher has retracted this article because an incorrect version was published in error. The final accepted version has now been published [1]. All authors agree with this retraction. Springer Nature apologises to the authors, Editors-in-Chief and readers for this error.
